# 2322: The effects of fecal microbiota transplantation on the gut microbiota in subjects with *Clostridium difficile* infection

**DOI:** 10.1017/cts.2017.130

**Published:** 2018-05-10

**Authors:** Amy Elizabeth Langdon, Christopher Bulow, Kim Reske, Sherry Sun, Tiffany Hink, Courtney Jones, Carey-Ann D. Burnham, Erik R. Dubberke, Gautam Dantas

**Affiliations:** 1 Washington University School of Medicine, St. Louis, MO, USA;; 2 Barnes Jewish Hospital, St. Louis, MO, USA;; 3 Rebiotix, Inc., Minneapolis, MN, USA

## Abstract

OBJECTIVES/SPECIFIC AIMS: *Clostridium difficile* is the most common cause of infectious antibiotic associated diarrhea. It is often refractory to antimicrobial therapy and fecal microbiota transplantation (FMT) is emerging as a therapeutic option. The objective is to characterize the direct effects of FMT on the gut microbiota. METHODS/STUDY POPULATION: Fecal specimens were obtained from a cohort of 29 subjects with recurrent *C. difficile* infection who received FMTs from 1 of 4 healthy donors as part of a phase 2 trial (Rebiotix). Fecal specimens were collected from the subject before FMT and up to 6 months post FMT. 16S rRNA sequencing and whole-genome shotgun sequencing were used to assess microbial community composition as compared by weighted Unifrac. RESULTS/ANTICIPATED RESULTS: Before treatment, the microbial community of subjects with *C. difficile* infection was highly distinct from the composition of the healthy donors in terms of metabolic profile. Quantification of phylogenetic community distance from donor by weighted Unifrac distance showed a significant decrease within the 1st week (Wilcoxon rank sum, *p*<0.01). This metric was predictive of both treatment failures and antibiotic resistance gene count (LR=22.45, *p*<0.0001). DISCUSSION/SIGNIFICANCE OF IMPACT: We conclude that distance from donor is a useful metric to quantify FMT success and that FMTs are a promising treatment for otherwise untreatable carriage of antibiotic resistance genes and organisms.

